# Doing no harm in mindfulness-based programs: Conceptual issues and empirical findings

**DOI:** 10.1016/j.cpr.2019.01.001

**Published:** 2019-07

**Authors:** Ruth Baer, Catherine Crane, Edward Miller, Willem Kuyken

**Affiliations:** a University of Kentucky, USA; b University of Oxford, UK

**Keywords:** Mindfulness, Mindfulness-based programs, Harm, Adverse outcomes

## Abstract

The benefits of empirically supported mindfulness-based programs (MBPs) are well documented, but the potential for harm has not been comprehensively studied. The available literature, although too small for a systematic review, suggests that the question of harm in MBPs needs careful attention. We argue that greater conceptual clarity will facilitate more systematic research and enable interpretation of existing findings. After summarizing how mindfulness, mindfulness practices, and MBPs are defined in the evidence-based context, we examine how harm is understood and studied in related approaches to physical or psychological health and wellbeing, including psychotherapy, pharmacotherapy, and physical exercise. We also review research on harmful effects of meditation in contemplative traditions. These bodies of literature provide helpful parallels for understanding potential harm in MBPs and suggest three interrelated types of factors that may contribute to harm and require further study: program-related factors, participant-related factors, and clinician- or teacher-related factors. We discuss conceptual issues and empirical findings related to these factors and end with recommendations for future research and for protecting participants in MBPs from harm.

## Highlights

• Potential harmful outcomes of mindfulness-based programs are under-researched.
• Harm occurs in psychotherapy, pharmacotherapy, physical exercise, and meditation.
• Potential harm may be related to participant, program, and instructor factors.
• Mindfulness practice can be unpleasant and challenging without causing harm.
• Understanding of harm in mindfulness programs requires monitoring individual data.

## 1 Introduction

Empirically supported mindfulness-based programs (MBPs) such as mindfulness-based stress reduction (MBSR; Kabat-Zinn, 1990) and mindfulness-based cognitive therapy (MBCT; Segal, Williams, & Teasdale, 2013) are widely used in healthcare, educational, and workplace settings. Meta-analytic reviews have found MBSR, MBCT, and closely related programs to have beneficial effects on a range of outcomes (Gotink et al., 2015). These include psychological disorders, stress, and coping with illness and pain (Khoury et al., 2013); positive moods and compassion for self and others (Khoury, Sharma, Rush, & Fournier, 2015); and some forms of attention and memory (Chiesa, Calati, & Serretti, 2011). Measurable effects on neural structures and systems have been documented (Tang, Hölzel, & Posner, 2015) and effects on blood pressure and immune function have been seen in some populations (Carlson, Speca, Faris, & Patel, 2007; Nyklíček, Mommersteeg, Van Beugen, Ramakers, & Van Boxtel, 2013). Comparisons with other interventions suggest that MBPs produce better outcomes than psychoeducation and support groups and comparable outcomes to cognitive-behavioral therapy and maintenance antidepressant medication (Goldberg et al., 2018; Kuyken et al., 2016).

Although the benefits of MBPs are well supported, less attention has been paid to potential harm. The study of harm in MBPs is essential for several reasons. First, any intervention powerful enough to have substantial benefits might also cause harm (Dimidjian & Hollon, 2010). In health-related professions, prevention of harm is the primary ethical duty and requires knowledge of harms that might occur and how to mitigate them. Second, psychotherapy researchers have long recognized that the study of harmful outcomes can lead to improved treatment methods (Dimidjian & Hollon, 2011; Mohr, 1995); the same is likely true for MBPs. Third, meditation as practiced in Buddhist traditions (e.g., Vipassana, Zen), can elicit challenging and difficult experiences, some of which can be serious and long lasting (Lindahl, Fisher, Cooper, Rosen, & Britton, 2017). It is therefore essential to ask whether similar effects might arise in evidence-based MBPs. Finally, popular media articles about “mindfulness” (often not clearly defined) sometimes suggest that it can be harmful (Foster, 2016). The scientific literature does not yet provide sufficient understanding of harm in MBPs to inform evidence-based perspectives on such articles. Thus, both the research literature and the public interest seem to require more systematic study of the potential for harm in MBPs.

In this paper, we discuss conceptual and empirical work from the scientific literature on mindfulness and related topics that bears on how to understand and investigate the harm that might arise for participants in evidence-based MBPs. Although too small for a systematic review, the literature suggests that the question of harm in MBPs needs careful attention, including greater conceptual clarity to facilitate more systematic research and interpretation of extant findings. After summarizing how mindfulness, mindfulness practices, and MBPs are defined in the evidence-based context, we examine how harm is understood and studied in related approaches to health and wellbeing, including psychotherapy, pharmacotherapy, and physical exercise. We also review research on harmful effects of meditation in contemplative traditions. These diverse bodies of literature provide helpful parallels for understanding potential harm in MBPs and suggest three types of factors that may contribute to harm: program-related factors, participant-related factors, and clinician- or teacher-related factors. We discuss empirical findings and conceptual issues related to these factors and end with recommendations for future research and for protecting participants in MBPs from harm.

## 2 Defining mindfulness, mindfulness practices, and MBPs in the evidence-based context

Evidence-based MBPs include ideas and practices adapted from Buddhist traditions. Although discussion continues about the relationship between the secular and the religious in MBPs (Brown, 2016; Compson, 2017), most are transparent about the Buddhist roots of mindfulness while aiming to be suitable for mainstream settings, accessible to diverse participants, and researchable within scientific disciplines related to health and wellbeing (Crane et al., 2017). To serve these aims, central ideas and practices in MBPs are conceptualized using contemporary scientific and discipline-specific language (Baer, 2011).

### 2.1 Mindfulness

In the scientific literature, mindfulness is usually defined as a form of present-moment attention and awareness that includes two elements: the attention itself and the qualities of the attention (sometimes described as the *what* and the *how* of mindfulness). Examples shown in Table 1 indicate that mindfulness is understood to be open, nonjudgmental, friendly, curious, accepting, compassionate, and kind. Mindfulness can be further conceptualized as a state in which these qualities of awareness are present, as a dispositional or trait-like general tendency to pay attention in these ways, and as a set of skills that develop with practice (Brown, 2016; Linehan, 1993). In all three of these forms, mindfulness has been shown to be correlated negatively with maladaptive psychological processes and positively with health and wellbeing (Levin, Hildebrandt, Lillis, & Hayes, 2012; Khoury et al., 2013; Quaglia et al., 2016).

**Table 1:** Contemporary psychological descriptions of mindfulness: *what* and *how.*

Author	*What*	*How*
Kabat-Zinn, 1994, Kabat-Zinn, 2003	Paying attention, or the awareness that arises through paying attention	on purpose, in the present moment, and nonjudgmentally; with an affectionate, compassionate quality, a sense of openhearted, friendly presence and interest
Marlatt & Kristeller, 1999	Bringing one's complete attention to present experiences	on a moment-to-moment basis, with an attitude of acceptance and loving-kindness
Bishop et al., 2004	Self-regulation of attention so that it is maintained on the immediate experience	with an orientation characterized by curiosity, openness, and acceptance
Germer, Siegel, & Fulton, 2005	Awareness of present experience	with acceptance: an extension of nonjudgment that adds a measure of kindness or friendliness
Linehan, 2015	The act of focusing the mind in the present moment	without judgment or attachment, with openness to the fluidity of each moment

### 2.2 Mindfulness practices

In evidence-based MBPs, mindfulness practices are exercises that cultivate the *what* and *how* elements of mindfulness. In most practices, participants are invited to focus their attention on present-moment phenomena, to notice when the mind wanders and return to the intended focus, and to bring an attitude of friendly curiosity and nonjudgmental acceptance to whatever is observed. In informal practices, these skills are applied to routine activities such as eating, walking and washing dishes. In formal practices, time is devoted solely to cultivating these skills. Some formal practices, such as sitting meditation, have roots in Buddhist traditions but are adapted for mainstream settings. Other practices were developed for more specific contemporary purposes. For example, in mindfulness-based childbirth and parenting (MBCP; Veringa et al., 2016), participants hold ice cubes for 60 s (the average length of a contraction) while attending mindfully to their breath and the sensations in their hands. This practice is intended to reduce the fear and stress associated with childbirth by teaching a new way of being present with sensations of pain.

### 2.3 Mindfulness-based programs

MBPs integrate theories and practices from contemplative traditions with the scientific disciplines of psychology, medicine, and education (Crane et al., 2017). They are based on a model of human experience that places ways of relating and responding to distress (rather than the distress itself) at the core of many problems and disorders. Through intensive training in formal and informal mindfulness practices and related exercises, MBPs teach a new relationship with present-moment experience based on approach (rather than avoidance), compassion, and decentering. The learning process is highly experiential. Mindfulness practices are followed by an interactive process known as inquiry that helps participants learn to identify their thoughts, emotions, and sensations, recognize habitual patterns of reacting to them, and respond with greater awareness and flexibility. Intended outcomes include attentional, emotional, and behavioral self-regulation as well as equanimity, compassion, and wisdom.

The first MBP to appear in the research literature was MBSR (Kabat-Zinn, 1982), an 8-week group program for adults with stress, pain, and health concerns. According to Kabat-Zinn (2011), one intention of MBSR was to recontextualize mindfulness within science, medicine, and healthcare “so that it would be maximally useful to people who could not hear it or enter into it through the more traditional dharma gates” (p. 288). MBSR is a skills training and psychoeducational program that uses formal and informal mindfulness practices suitable for contemporary non-Buddhist settings.

In defining the essential features of MBPs, Crane et al. (2017) include programs derived from or inspired by MBSR, such as MBCT, MBCP, mindfulness-based relapse prevention (Bowen et al., 2009), mindfulness-based eating awareness training (Kristeller, Wolever, & Sheets, 2014), and others, but not programs such as dialectical behavior therapy (DBT; Linehan, 2015) and acceptance and commitment therapy (ACT; Hayes, Strosahl, & Wilson, 2012). DBT and ACT integrate mindfulness exercises with a variety of other therapeutic strategies, place less emphasis on formal meditation, and are generally identified as forms of psychotherapy, whereas MBPs are often described as educational and skills training programs. For these reasons, this paper addresses the MBSR-inspired family of MBPs but not DBT, ACT, or other psychotherapies with mindfulness elements.

## 3 The study of harm in related fields

Little is known about the potential for harmful outcomes in MBPs. In contrast, other approaches to health and wellbeing, including psychotherapy, pharmacotherapy, and physical exercise, have examined the issue in more detail. In the following sections, we summarize research on harm in these fields and draw parallels that may be helpful in understanding how the risk of harm from MBPs can be conceptualized and studied. We also summarize recent work on the potential for harm from meditation in contemplative traditions and consider its applicability to the question of harm in MBPs.

### 3.1 Psychological treatment

Duggan, Parry, McMurran, Davidson, and Dennis (2014) define harm as “a sustained deterioration that is caused directly by the psychological intervention” (p. 2). Dimidjian and Hollon (2010) state that harmful psychological treatments are damaging and injurious and cause worse outcomes than would have occurred without treatment. The potential for psychotherapy to cause harm has been recognized for many years (Bergin, 1966). Large scale studies and reviews have consistently concluded that between 3% and 10% of psychotherapy clients get worse with treatment (Lambert, 2013; Mohr, 1995; Strupp, Hadley, & Gomez-Schwartz, 1977). For example, in a sample of over 6000 psychotherapy clients in the US, Hansen, Lambert, and Forman (2002) found that 8.2% showed reliable deterioration on a well-validated outcome questionnaire. Crawford et al. (2016) surveyed over 75,000 recipients of psychological treatment through the National Health Service in England and Wales and found that, of the 14,587 who returned the survey, 763 (5.23%) agreed (slightly or strongly) that they had experienced “lasting bad effects from the treatment.”

Harm in psychotherapy can take many forms. The target problem may get worse or a new problem may arise while the target problem improves or remains unchanged (Dimidjian & Hollon, 2010). Linden (2013) proposed definitions for several unwanted outcomes of psychotherapy including adverse reactions (unwanted events caused by the treatment), side effects (unwanted events caused by an effective treatment), malpractice effects (unwanted events caused by inappropriate treatment), and contra-indications (serious side effects rendering a treatment inappropriate for some people). Linden and Schermuly-Haupt (2014) further noted that unwanted events can be mild (no consequences), moderate (distressing), severe (in need of countermeasures), very severe (lasting negative consequences), or extremely severe (hospitalization required or life threatening). Mild-to-moderate events should be understood and avoided when possible but do not appear to meet definitions of harm, whereas events higher in the scale are clearly harmful.

When deterioration over a course of therapy is observed, its causes may not be clear. In some cases, the deterioration might not be attributable to the treatment, which may be inert or may slow but not reverse an ongoing worsening of symptoms. Even so, Lambert (2013) noted that when deterioration is monitored in controlled studies, it is often worse in treatment groups than in no-treatment controls, suggesting that aspects of treatment may be responsible. Empirical work on harm in psychotherapy has examined three types of variables that are correlated with negative outcomes: client, therapist, and treatment variables.

Client variables related to negative outcomes include severity of symptoms, poor interpersonal skills (Mohr, 1995), diagnostic comorbidity, severe stressors (Dimidjian & Hollon, 2011), and demographic variables. Crawford et al. (2016) found that clients older than 65 years were less likely to report negative effects whereas sexual and ethnic minorities were more likely to report them. Therapist variables include lack of skill in conducting an effective therapy (Lilienfeld, 2007), lack of empathy, underestimating the severity of the client's problems, and poor communication about the process and content of therapy (Crawford et al., 2016; Mohr, 1995). Treatments themselves can also be harmful. Lilienfeld (2007) identified therapies shown in randomized trials to produce worse outcomes than comparison groups. For example, critical incident stress debriefing can worsen symptoms of post-traumatic stress (Litz, Gray, Bryant, & Adler, 2002), perhaps by interfering with natural processes of recovery. Boot-camp approaches to conduct problems in adolescents may cause increases in criminal behaviour (Weiss, Wilson, & Whitemarsh, 2005.

### 3.2 Pharmacotherapy

Adverse drug reactions (ADRs) are defined as appreciably harmful or unpleasant reactions to medications (Aronson & Ferner, 2005) resulting from error, misuse, and off-label use, as well as authorised use in normal doses (Coleman & Pontefract, 2016). ADRs are occasionally fatal and often uncomfortable, costly, and damaging to the patient-prescriber relationship. Incidence of ADRs has been estimated at 5–10% of hospitalized patients (Lazarou, Pomeranz, & Corey, 1998). The three types of variables related to harm in psychotherapy are also recognized in pharmacotherapy. Patient factors and drug factors are often discussed in terms of pharmacokinetics and pharmacodynamics, or “what the body does to the drug” and “what the drug does to the body” (Meibohm & Derendorf, 1997, p. 401). Pharmacokinetics includes how the body absorbs, distributes, metabolizes, and excretes the drug; pharmacodynamics, are the biochemical and physiological effects of the drug on the body. Drug effects, therefore, are mediated by a complex interaction of drug factors and patient factors, including dosage and frequency of administration, the patient's genetic profile, the presence of other drugs in the body, and tolerance to the drug.

Clinician factors are also important. Clinicians must identify patients who are likely to be susceptible to ADRs, modify the treatment choice accordingly, and include in the treatment plan strategies for mitigating ADRs that arise (Coleman & Pontefract, 2016). They must explain the risks and benefits of taking or not taking the drug and alternative options. For patients who choose to take the drug, failure to take it correctly can cause harm. Bosworth et al. (2011) noted that thousands of deaths and hospitalizations each year are attributable to medication nonadherence. A large literature suggests that clinicians can reduce the risk of harm from nonadherence through information, advice, and counselling that increase patients' ability and willingness to take medication as prescribed (Schulz, 2007).

### 3.3 Physical exercise

Mindfulness is sometimes compared to physical exercise, with analogies to training the attention through mindfulness exercises “rather in the same way that we go to a gym to train muscles” (Segal et al., 2013, p. 97). The benefits and harms of physical exercise are well researched. The American College of Sports Medicine (Garber et al., 2011) notes that the benefits far outweigh the risks in most adults and include reduced mortality from many causes, prevention of chronic medical conditions, reductions in anxiety and mood disorders, and improved wellbeing and quality of life (Warburton, Taunton, Bredin, & Isserow, 2016). The most common risks are musculoskeletal, including strains, sprains, tears, inflammation, and fractures. More serious risks are cardiovascular, including arrhythmias, heart attacks, and sudden cardiac arrest. Conn, Annest, and Gilchrist (2003) reported that 2.59% of Americans annually receive medical attention for a sports or recreation-related injury. The risk of a cardiovascular event linked to aerobic exercise varies with fitness level and falls between 1 in 500,000 and 1 in 2,600,000 h of exercise (Franklin & Billecke, 2012).

As in psychotherapy, risks of physical exercise are often discussed in terms of participant, program, and instructor factors. Participant factors include age, health status, physical activity, and fitness. Program factors include intensity of the exercise, tailoring to individual participants, screening procedures, and education about risks. Instructor factors include knowledge of the physiology of exercise, competence in screening participants, adapting programs to individuals, and encouraging adherence (Garber et al., 2011). These factors interact in interesting ways to influence risk/benefit ratios. For example, the dose-response relationship between exercise and health status is not linear. For inactive people, small increases in exercise lead to substantial health benefits (Lollgen, Bockenhoff, & Knapp, 2009). In contrast, extremely active people may experience diminishing returns for health benefits as well as cardiovascular damage (Patil et al., 2012). Warburton et al. (2016) note that people engaged in intensive training for “ultra-endurance events” are at increased risk for cardiovascular disease and “should be cautioned about the perils involved” (p. 216).

### 3.4 Meditation in contemplative traditions

Case reports of one or a few individuals describe severe symptoms induced by meditation, including psychosis (Kuijpers, Van der Heijden, Tuinier, & Verhoeven, 2007), negative affect (French, Schmid, & Ingalls, 1975), mania (Yorston, 2001), depersonalization and derealization (Castillo, 1990), and traumatic memories (Miller, 1993). Such outcomes have been associated with several types of meditation (transcendental, Zen, mindfulness) and often occurred in the context of intensive retreats (Lustyk, Chawla, Nolan, & Marlatt, 2009). Most of these early studies did not address the prevalence of harmful outcomes in meditating samples or account for pre-existing psychological difficulties. None involved evidence-based MBPs.

Larger studies reporting percentages of meditating samples with negative experiences are summarized in Table 2. Otis (1984) found that 4.5% - 13.5% of transcendental meditation practitioners (*N* = 574) reported increases in anxiety, depression, confusion, and other symptoms. The more experienced meditators reported more symptoms, but also more psychological problems prior to taking up meditation. In long-term Vipassana practitioners (*N* = 27), Shapiro (1992) found that while most reported more positive than negative effects, 63% reported at least one challenging experience such as confusion, alienation, or negative emotion. Some described these experiences as learning opportunities rather than problems; however, two (7%) reported severe effects (disorientation and depression) that caused them to stop meditating. In an internet survey of 342 meditation practitioners (Cebolla, Demarzo, Martins, Soler, & Garcia-Campayo, 2017), 25.4% reported unwanted effects (UEs) including negative emotions, pain, depersonalization, and other symptoms. Many of these symptoms were described as transitory, although missing data were extensive. In 1.1% of the sample, UEs caused the person to stop meditating; 5.7% sought help from a medical professional or therapist. Positive outcomes were not addressed.

**Table 2:** Studies reporting percentages of meditating samples (none from MBIs) describing negative or unwanted effects of meditation.

1st author, year	Sample	Percent reporting and types of negative effects	Comments
Otis, 1984	574 TM practitioners	4.5% - 13.5% reported anxiety, depression, confusion, or other symptoms	More experienced meditators reported more negative effects and more problems prior to taking up meditation
Shapiro, 1992	27 long-term Vipassana meditators	63% reported negative emotion, confusion, alienation, or other symptoms 7% reported severe effects (disorientation, depression) that led them to stop meditating	Many described unpleasant experiences as temporary and as learning opportunities Over 80% reported positive outcomes (joy, confidence, acceptance, compassion, problem solving, resilience) Psychiatric history not addressed
Cebolla, 2017	342 practitioners of many types of meditation	25.4% reported unwanted events (anxiety, pain, mood symptoms, other) 1% stopped meditating 5.7% sought professional help	Many described unwanted events as transitory Positive effects not addressed Psychiatric history not reported Extensive missing data
Lomas, 2015	30 male Buddhist meditators	100% described meditation as challenging (difficult, unpleasant thoughts and emotions) 25% of the interview data involved problems with meditation 20% reported threats to sense of reality 7% hospitalized (1 suicidal)	100% described meditation as valuable and conducive to wellbeing Many described difficulties (even severe ones) as important to their development

More detailed accounts are provided by Lomas, Cartwright, Edginton, and Ridge (2015), who interviewed 30 male Buddhist meditators about the impact of meditation on their wellbeing. All were regular practitioners of various types of meditation for durations ranging from less than five to >20 years. Three were receiving mental health treatment at the time of the interview; 11 had done so in the past. All described meditation as a valuable activity and conducive to wellbeing. However, reports of substantial difficulties accounted for about one quarter of the interview data and these reports became the subject of the paper.

For example, most participants reported that meditation brought up troubling thoughts and feelings that were hard to manage. Many stated that depression, anxiety, and low self-esteem were exacerbated by meditation. Six reported serious threats to their sense of reality; for example, they felt unreal, disoriented, or alienated. The authors note that “these episodes did not occur in relation to more conventional practices like mindfulness or loving-kindness meditation” but arose when “attempting advanced meditation practices while still being a relative beginner” and doing so “without the guidance of an experienced teacher and/or a supportive sangha” (p. 855). The most severe difficulties were psychotic symptoms. One participant felt close to psychosis when trying to resume normal life following a week of intensive practice in isolation. Two others were hospitalized for psychotic episodes; one of them, who was also suicidal, attributed this to meditation. Five of the six who reported threats to their sense of reality reported no mental health problems before starting meditation and four attributed these experiences directly to the meditation practice. Despite the difficulties, many of the participants said that they eventually learned skills for managing them and came to see such experiences as important to their wellbeing and psychological development. The authors concluded that meditation can have substantial positive and negative effects.

Overall, the studies in Table 2 suggest that unpleasant and difficult experiences in meditation are common. In three of the four studies, many participants described these experiences as temporary and useful in developing skills and insights. Quantitative data are not always provided, making it hard to determine the prevalence of difficulties that outweigh benefits. However, severe and harmful effects were reported, with 1% - 7% of participants quitting meditation, seeking professional help, or being hospitalized.

In the most detailed qualitative study to date of meditation-related harm, Lindahl et al. (2017) interviewed 60 Western Buddhist practitioners from the Zen, Theravada, and Tibetan traditions (57% male, mean age = 49 years, all residing in North America or Europe). Participants were eligible only if they reported difficult, challenging, distressing, or impairing experiences related to their meditation practice that could not plausibly be attributed to pre-existing psychological or medical conditions or other factors. Thus, this study provides no information about base rates of difficulties in meditating samples and is not included in Table 2. Most participants were White and held university degrees; 60% were meditation teachers. For 72%, meditation-related difficulties began during or shortly after a retreat. For 28%, they were associated with daily practice. None had participated in evidence-based MBPs.

Semi-structured interviews yielded 59 categories of meditation-related effects that were clustered into seven domains: cognitive (change in executive functioning, delusions), perceptual (hallucinations, distortions in time or space), affective (positive or negative affect), somatic (pain, energy, sleep, movement-related), conative (motivation-related), sense of self (self-other or self-world boundaries, sense of agency) and social (social impairment, change in relationships). Most (73%) reported moderate to severe impairment in at least one domain, 17% reported suicidality, and 17% required inpatient hospitalization. Median duration of impairment was 1–3 years, with a range of a few days to >10 years.

Analyses also yielded four types of factors potentially related to difficult meditation experiences, corresponding loosely to the three factors identified in the previous sections (participant, program, and clinician/instructor factors). Participant factors included psychiatric, medical, and trauma history, motivations or goals for meditating, worldview, and personality. Health behavior factors (which can also be seen as participant factors) included diet, sleep, exercise, and use of medications or recreational drugs. Practice factors included the amount, intensity, consistency, type, and stage of practice. Relationship factors included relationships with teachers, practice community, and others, as well as their early life relationships. Teacher factors other than relationship with the meditator were not discussed.

A striking finding of this study was the lack of consistency among participants in whether specific meditation effects were seen as adverse. Many intense experiences, including affect, pain, and paranoia, were appraised in a variety of ways. The authors note that interpretive frameworks in the Buddhist traditions are diverse, with differences “across traditions, lineages, or even teachers” in whether specific meditation-related experiences should be seen as “progress” or “pathology” (p. 25). Differences between Buddhist and psychological or medical perspectives are also evident. Experiences that seem pathological from a clinical point of view (hallucinations, paranoia) might not be viewed as harmful if understood by the practitioner and teacher as transitory experiences that make sense within the conceptual framework of their meditation tradition and can be managed constructively.

Participants in this study were recruited through “outlier sampling” (p. 7) and represent the extreme adverse end of the distribution of meditation-related effects. This was intentional, as the purpose was to study under-reported phenomena; however, it prevents conclusions about the base rates of distressing or impairing effects in the overall population of Buddhist meditation practitioners. The extent to which findings apply to participants in MBPs is hard to determine. Although 25% of participants who reported harm were practicing less than one hour per day using practices similar to those in MBPs and for similar purposes (mental health-related rather than spiritual purposes), they may not have had the psychoeducational and structural supports typical of MBPs (Lomas et al., 2015). We discuss these supports in more detail in a later section.

### 3.5 Harm in related fields: parallels for the study of evidence-based MBPs

The literature just reviewed suggests several parallels for the study of harm in evidence-based MBPs. First, interventions with established benefits can also cause harm. Between 3 and 10% of psychotherapy clients get worse, 5–10% of hospitalized patients have adverse drug reactions, a small percentage of participants in physical exercise are injured, and an unclear percentage of meditators in contemplative traditions experience harmful effects. MBPs include meditation and are provided in both psychotherapeutic and wellness contexts; it therefore seems likely some participants may experience harmful outcomes.

Second, sources of potential harm in the four fields just reviewed can be classified into three categories that may also apply to MBPs: 1) program, treatment, or practice factors, 2) client, patient, or participant factors, and 3) teacher, therapist, or clinician factors. Examples (not an exhaustive list) are shown in Table 3. Although it is useful to consider these factors separately, in many situations they probably work together. For example, some medications are hard to take correctly (treatment factor); e.g., those that must be taken several times daily under specific conditions. For a patient whose circumstances make this difficult or whose personality is low in conscientiousness (patient factors), help from the prescriber (clinician factor) with problem-solving or finding a different medication may be necessary. An example from psychotherapy is relaxation-induced anxiety (Ferguson & Sgambati, 2008), which may be related to fear of losing control (participant factor) and can be addressed by trying a different form of relaxation (program factor) or providing enhanced explanation and guidance (therapist factor). In both of these examples, skillful reduction of the likelihood of harm integrates participant, program, and clinician factors. The same is likely true of MBPs.

**Table 3:** Sources of harm in related approaches to health and wellbeing.

Discipline	Program/intervention factors	Participant factors	Teacher/clinician factors
Psychotherapy	theoretically unsound, interferes with natural psychological processes, wrong treatment for presenting problem	symptom severity, comorbidity, poor interpersonal functioning, severe psychosocial stressors	lack of empathy, underestimating severity of client's problems, lack of clarity about process or content of therapy, other lack of competence
Pharmacotherapy	dosage, frequency of administration, pharmacodynamics	genetic profile, other drugs in body, pharmacokinetics, nonadherence	lack of knowledge of drug effects, lack of skills for encouraging adherence
Physical exercise	not tailored for individual, too intense, lack of screening or education about risks	age, health status, fitness level, physical activity	lack of general competence, lack of skills for encouraging adherence
Meditation in contemplative traditions	amount, intensity, consistency of practice; type or stage of practice	psychiatric, medical, or trauma history; goals for practice, personality, health habits, relationships	relationship with practitioner

The dose-response relationship and adherence to recommendations, which have been studied in pharmacotherapy, physical exercise, and psychotherapy, may also be important in evidence-based MBPs. Whether higher doses of meditation can be harmful, within the range recommended by MBPs, is unknown. Whether lack of adherence in MBPs can cause harm (rather than unhelpfulness) through under- or overdosing is also unknown. Clinician or instructor factors, such as educating participants about rationales, risks, and benefits, and tailoring programs for individual needs, may also be important in preventing harm in MBPs.

Finally, it seems clear that many approaches to health and wellbeing can be stressful and challenging. Effective medications can have unpleasant side effects. Physical exercise can cause soreness and fatigue. Difficult experiences in meditation are sometimes seen as learning opportunities or indications of progress along the meditative path. Temporary discomfort also seems to be inevitable in psychotherapy (Duggan et al., 2014) as participants confront painful issues, learn new skills, and apply them in problematic situations. In the next section we consider the important distinction between lasting harm and the discomfort associated with psychological change. We focus on evidence-based psychotherapies, where discomfort has been widely discussed, and then consider how the issues apply to MBPs.

## 4 Stress and discomfort in psychological change

Many evidence-based psychological treatments include difficult activities. Behavioral experiments, for example, are designed to test unhelpful beliefs, such as, “If I participate in conversation, I'll say stupid things and people will reject me.” Testing these beliefs by engaging in such behavior, even in carefully planned ways, is expected to induce anxiety (Bennett-Levy et al., 2004). In fact, behavioral experiments that induce too little anxiety tend to be ineffective because they create too little change in clients' beliefs about what they can do. An effective behavioral experiment is a “challenge to prevailing perspectives” and likely to seem “at least somewhat threatening” (Bennett-Levy et al., 2004; p. 43). The same applies to exposure-based therapies. A client with obsessive-compulsive disorder may be encouraged to touch a dirty floor and then refrain from handwashing, a person with post-traumatic stress to recount a traumatic experience in detail. Many clients find these procedures challenging.

Some mental health professionals believe that exposure-based methods cause attrition or exacerbation of symptoms and that clients are better off continuing with the disorder than participating in the treatment (Becker, Zayfert, & Anderson, 2004; Richard & Gloster, 2007). These concerns are inconsistent with the strong empirical evidence supporting the efficacy of exposure-based therapies (Olatunji, Deacon, & Abramowitz, 2009). Deacon and Abramowitz (2005) reported that people with anxiety disorders perceive exposure-based CBT as more acceptable than medications and more likely to be effective in the long term. Foa, Zoellner, Feeny, Hembree, and Alvarez-Conrad (2002) found that prolonged exposure for PTSD caused temporary worsening of symptoms in a minority of participants, but that this short-term effect did not increase the risk of attrition or reduce the benefits attained by the end of treatment.

Recommendations for avoiding harm in such treatments include: (a) remembering that many clients are “vulnerable people whose confidence is readily shaken” (Bennett-Levy et al., 2004, p. 33), (b) providing a clear rationale for and explanation of the activity, (c) never coercing a client to engage in the procedure, but encouraging the client to collaborate in designing the exercises and make their own decisions about participating in them, (d) using tasks that objectively are no more risky than daily life (e.g., asking a stranger for directions may feel terrifying to a client with severe social anxiety, but is generally not dangerous), and (e) anticipating how the activity might go awry and how this will be interpreted and managed. Olatunji et al. (2009) note that the potential for discomfort makes exposure-based therapies complex to implement and that their most substantial risk may be unskillful delivery by therapists with inadequate training and supervision.

### 4.1 Stress and discomfort in MBPs

Participation in MBPs also involves discomfort. Unwanted thoughts, emotions, and sensations inevitably arise during practices. Segal et al. (2013) recommend discussing these difficulties during a pre-class interview and providing a clear rationale for how the program may help with participants' concerns. During the course, teachers often remind participants not to push beyond limits of safety or tolerance and suggest ways to adapt practices if difficulties that feel overwhelming arise.

Within these limits, however, discomfort is generally approached (with compassion) rather than avoided. In MBCT, a handout explains that mindful awareness in daily life means “facing what is present, even when it is unpleasant and difficult” (p. 102) and that learning to do this gently, with the support of the teacher and the group, is “the most effective way, in the long run, to reduce unhappiness.” To cultivate acceptance skills, several MBPs include a practice in which participants are invited to bring a problem to mind and observe the associated thoughts and feelings with friendly curiosity. Although no studies have examined the effects of this practice separately from the rest of the curriculum, an extensive body of research shows the maladaptive effects of avoidance and suppression of thoughts, emotions, and sensations and the benefits of accepting them as they are (Cameron & Overall, 2018; Hayes, Wilson, Gifford, Follette, & Strosahl, 1996; Kashdan & Rottenberg, 2010; Levin et al., 2012). Clinical observations suggest that learning skills for facing difficulties is empowering (Sears, 2015).

### 4.2 Weighing the risk of discomfort against the potential for benefit

In their discussion of uncomfortable but effective therapies, Olatunji et al. (2009) noted that the risks of engaging in them must be balanced against the risks of not doing so. If alternative treatments are less effective, the risk that symptoms will continue may be worse than the temporary discomfort caused by the treatment. The balance between acceptable discomfort and potential benefit may vary with the severity of the participant's problems. A person with a severe disorder probably feels much distress in daily life and may find that difficult therapeutic exercises, though uncomfortable, are within the range of discomfort caused by their symptoms and worthwhile in light of the likely benefits. In contrast, a psychologically healthy person who takes a mindfulness course for personal growth and unexpectedly has an intensely uncomfortable experience may find the discomfort disproportionate to the expected benefits. Such discomfort may not qualify as harm, by current definitions, if it doesn't lead to sustained deterioration. If the difficult experience is disclosed, a skilled teacher may be able to help the participant work with it in beneficial ways. On the other hand, the difficult experience may be sufficiently distressing to qualify as an adverse event. This term is defined in the next section.

## 5 Harm and adverse events in MBPs: Definitions and current findings

We suggest that the most useful definition of harm in MBPs is based on the definitions used in psychotherapy. That is, after exposure to the MBP (whether the participant completes it or drops out), harm has occurred if the participant's symptoms or level of functioning are worse than beforehand and this deterioration is sustained, attributable to the program, and more severe than it would have been without the program. As in psychological treatment, harm in MBPs can appear in a variety of ways. We agree with Dimidjian and Hollon (2010) that harm is worse than unhelpfulness. An unhelpful program confers no benefit and will not stop an ongoing process of deterioration, whereas a harmful program makes matters worse than they would have been otherwise.

The terms *adverse events* (AEs) and *serious adverse events* (SAEs) are often used in healthcare research and have been adopted in some studies of MBPs. As defined by the World Health Organization, an AE is an untoward occurrence in a patient or research participant who is administered a pharmaceutical product (Lineberry et al., 2016); an SAE is such an event that threatens life or function (Ioannidis et al., 2004). In behavioral health and psychotherapy trials, SAEs include events such as suicidal behavior and psychiatric hospitalizations; AEs are less severe changes in psychological, behavioral, or physical functioning (Peterson, Roache, Raj, & Young-McCaughan, 2013). By definition, AEs and SAEs are not necessarily caused by the intervention. The causes of these events can be difficult to determine. In clinical trials, AEs and SAEs are monitored in treatment and control groups; a higher frequency in the treatment group suggests that the treatment may be harmful.

A few trials have reported deterioration for participants in MBPs. For example, Reynolds et al. (2017) found symptom increases in an MBP for cancer patients. Johnson et al. (2016), in a school-based study, reported worse post-treatment anxiety scores in the MBP than in no-treatment for several subgroups of participants. Brooker et al. (2012), in an uncontrolled study of work-related stress, reported mixed findings, with deterioration on some outcome variables. These studies must be seen in the context of meta-analyses concluding that most studies support the benefits of MBPs for these populations, including children and adolescents (Dunning et al., 2018), cancer patients and survivors (Piet, Würtzen, & Zachariae, 2012), and workers in various occupations (Lomas, Medina, Ivtzan, Rupprecht, & Eiroa-Orosa, 2018).

We found four reviews addressing AEs and SAEs in MBPs; these are summarized in Table 4. Goyal et al. (2014) reviewed 47 RCTs comparing a mindfulness or meditation-based program to an active control condition. Of these, 19% (9 studies) reported on AEs; none of these reported any harms. In a review of 12 studies of MBPs for post-traumatic stress, Banks, Newman, and Saleem (2015) found that two studies did not report on AEs/SAEs, four reported that none occurred, and six reported that some participants showed increases in symptoms that were not clinically significant. Collapsed across these six studies, symptom increases occurred in 13 of 123 participants (10.6%). In two studies, a few participants reported increased anxiety during meditation practices but no significant worsening of symptoms from pre- to post-treatment. One participant reported that meditation triggered a memory of an assault. Learning to work skilfully with such memories was considered part of the treatment. The authors concluded that adverse effects in these studies were minimal.

**Table 4:** Reviews of evidence-based MBPs that include data on adverse events.

First author, year	Number and type of studies in the review	Percentage of studies in the review that reported data on AEs/SAEs	Findings for AEs/SAEs	Comments
Goyal, 2014	47 RCTs comparing MBPs or other meditation-based programs to active controls	19%	None reported	
Banks, 2015	12 studies (various designs) of MBPs for PTSD	83%	AEs in 10.6% of participants	symptom increases not clinically significant, anxiety during meditation practices did not lead to pre-post deterioration, 1 trauma memory triggered seen as within purpose of intervention
Kuyken, 2016	9 RCTs of MBCT for depressive relapse	56%	SAEs in 1.94% of participants (range: 0 to 5.5%)	SAEs no more common in MBCT than in control groups; SAEs unrelated to participation in MBCT
Wong, 2018	231 RCTs of MBSR or MBCT	16%	AEs in 1% of MBP participants, 0.9% of control participants	AEs no more common in MBPs than in control groups

A meta-analysis of nine RCTs of MBCT for relapse prevention in recurrent depression (Kuyken et al., 2016) reported that four of the trials included no data on AEs or SAEs. In the other five trials, only SAEs were reported. Percentage of participants in whom SAEs occurred ranged from zero (2 studies) to 5.5% (1 study) with a mean of 1.94%. SAEs were no more common in MBCT groups than in control arms of the trials and none were judged to be related to participation in MBCT. These studies did not report on broader indications of harm, such as worsening of symptoms, appearance of new symptoms, or general decline in functioning or wellbeing. Finally, a systematic review of RCTs of MBSR and MBCT (Wong, Chan, Zhang, Lee, & Tsoi, 2018) found that 195 of 231 trials (84%) did not report on AEs. When summed across the other 36 trials, AEs occurred in 1.0% and 0.9% of participants in MBI and control groups, respectively; these proportions were not significantly different. The authors concluded that MBSR and MBCT appear to be relatively safe but strongly recommended more consistent reporting of AEs and SAEs.

Qualitative studies of difficulties in MBPs suggest that they are common but tend to be short-term. A meta-ethnography (Malpass et al., 2012) reported that increases in participants' awareness of their maladaptive coping habits could feel temporarily overwhelming, that present-moment awareness was occasionally frightening, and that mistaken expectations that mindfulness should rid the mind of all depressive thoughts led to self-devaluation when this did not happen. Despite these difficulties, the general pattern was a shift from maladaptive coping strategies to more adaptive forms of self-understanding.

Overall, findings show that when reported, AEs/SAEs have occurred in 0 to 10.6% of participants in evidence-based MBPs. When compared in MBPs and controls, AEs/SAEs occur at similar rates, suggesting that they are unrelated to participation in the MBP. These findings must be interpreted cautiously for several reasons. First, few studies have report on AEs/SAEs. Second, AEs/SAEs are sometimes narrowly defined, such that general deterioration or the appearance of new symptoms might not be included. Finally, although meta-analyses and qualitative studies show strong evidence of improvement with MBPs, it remains unclear whether group averages mask deterioration in some participants.

## 6 Potential sources of harm in MBPs

As noted earlier, sources of harm commonly discussed in other approaches to health and wellbeing include program factors, participant factors, and teacher/clinician factors. In the following sections, we review empirical findings and conceptual discussion of how these sources may apply to MBPs.

### 6.1 Program factors

Here we address factors that have been studied or discussed in the mindfulness literature: the importance of *what* and *how* elements of mindfulness, the soundness of the programs' conceptual foundations, the intensity of the mindfulness practices, and the adequacy of the psychoeducational or structural support provided by the program.

#### 6.1.1 What and how

Segal et al. (2013) state that mindfulness “cannot be reduced to awareness or attention alone” and note that increased present-moment awareness will not be helpful, and may even be harmful, unless “friendliness and compassion can be brought to those elements of present-moment experience to which we attend” (p. 137). In support of this statement, several studies have found that the relationship between present-moment awareness and adaptive functioning is moderated by the quality of the awareness. Substance use, depression, rumination, worry, and blood pressure have all been shown to be lower in participants who endorse high levels of present-moment awareness, but only if the awareness is nonjudgmental or nonreactive (Desrosiers, Vine, Curtiss, & Klemanski, 2014; Eisenlohr-Moul, Walsh, Charnigo, Lynam, & Baer, 2012; Tomfohr, Pung, Mills, & Edwards, 2015). This work is consistent with previous research showing that self-focused attention (defined as awareness of thoughts, emotions, and sensations) is adaptive when it is nonjudgmental and experiential but maladaptive when it is judgmental and ruminative (Ingram, 1990; Mor & Winquist, 2002; Watkins, 2008). These bodies of literature suggest that effective teaching of mindfulness must include both present-moment awareness and its nonreactive, nonjudgmental qualities. Otherwise, increases in awareness might lead to unintended increases in symptoms.

#### 6.1.2 Conceptual foundations of the MPB

Crane et al. (2017) argued that without a sound theoretical formulation of how mindfulness should help with a particular problem, an MBP might be unhelpful. An interesting example to which this may apply is insomnia. Ong, Ulmer, and Manber (2012) theorize that mindfulness promotes decentering from insomnia-related thoughts and feelings, equanimity and commitment to values, and reduced sleep-related arousal. This model was supported in randomized trial showing improvements in self-reported sleep variables for people with insomnia (Ong et al., 2014). On the other hand, Britton, Lindahl, Cahn, Davis, and Goldman (2014) note that in Buddhist traditions, mindfulness is described as a state of relaxed alertness that balances hyper- and hypoarousal. They summarize brain imaging studies suggesting that “Buddhist meditation practices are associated with activation/enlargement of the areas that underlie tonic alertness and/or prevent sleep” (p. 69). A study of MBCT for people with partially remitted depression and sleep disturbance (Britton, Haynes, Fridel, & Bootzin, 2010) found polysomnographic evidence of increased cortical arousal post-MBCT that was correlated with amount of mindfulness practice, although participants also reported better subjective sleep quality. Britton et al. (2014) suggested that short-term, short-duration mindfulness practice may increase sleep propensity whereas longer-term, higher-dose practice may lead to neurological changes producing greater wakefulness. They did not discuss harm, but findings suggest that intensive long-term practice might be unhelpful for people whose goal is increased sleep. These findings highlight the necessity of a clear understanding of how mindfulness meditation, as used in MBPs, should be expected to improve sleep.

#### 6.1.3 Intensity of the mindfulness practice

Though loosely analogous to intensity of physical exercise and dosage of medication, intensity of mindfulness practice is hard to define. Participants in MBPs may have different experiences of the intensity of practice, perhaps related to previous meditation experience, the environment in which they practice, or other factors. The idea that intensity of practice could be related to harm in MBPs comes most directly from Lindahl et al. (2017), who reported that for 72% of their Buddhist practitioners, harmful effects were associated with participation in residential retreats, which are often a week or more in duration and involve many hours per day of meditation in a mostly silent environment removed from normal daily routines.

Most MBPs involve weekly group sessions and encourage up to an hour per day of formal and informal home practice. Many also include a mostly silent all-day session of about 6 h. Whether this level of intensity can cause harmful outcomes is unclear. Lindahl et al. reported that 25% of their participants who reported harm were practicing less than an hour per day using practices similar to those in MBPs and for similar purposes (mental health-related rather than spiritual purposes); accordingly, they argued that the harmful effects they observed may also occur in MBPs. While we agree that this possibility should be studied, we also note that evidence-based MBPs include many psychoeducational and structural supports that may be less available in Buddhist meditation settings where participants are practicing outside of monitored interventions (Lomas et al., 2015). These supports may reduce the potential for harm from challenging meditation experiences.

#### 6.1.4 Psychoeducational and structural support for meditation practices

Many MBPs include a pre-course interview or information session that helps participants anticipate and prepare for likely challenges. A rationale for how mindfulness is expected to help with participants' problems is often provided. Nearly all in-session practices are followed by inquiry, when challenging experiences can be explored. Sessions also include inquiry about home practice, when difficulties encountered at home can be considered. Teachers are often available before and after sessions for consultation. A range of practices, varying in duration and focus, is introduced in a logical sequence in which learning builds from week to week. Recordings to guide home practice are provided. Sessions also include didactic information and non-meditative exercises that supplement and support the meditation practices; many of these speak directly to how to manage challenging experiences that arise in meditation or at other times. Weekly handouts provide summaries of session content and goals, rationales for the practices, descriptions of previous participants' experiences, and worksheets for recording observations. The all-day session occurs late in the eight-week course, allowing participants to build skills through several weeks of daily practice and group sessions beforehand.

We identified only one example of empirical study of the effects of psychoeducational and structural supports. In a pilot trial for a study of MBCT adapted for people at risk of suicide, Crane and Williams (2010) found that participants who showed the greatest evidence of cognitive vulnerabilities associated with depression and suicidality were most likely to drop out. In the subsequent trial, the individual pre-course interview was extended from 60 to 90 min to include more explicit, personalised discussion of how to meet the program's likely challenges. Use of the modified interview reduced attrition from 30% to 18% (Williams et al., 2015), suggesting that evidence-based structural and psychoeducational support for meditation may be helpful in high-risk samples.

### 6.2 Participant factors

Table 2 suggests participant factors that could be related to the potential for harm in MBPs. The mindfulness literature has focused primarily on psychiatric and trauma history. Of the Buddhist meditation teachers interviewed by Lindahl et al. (2017), 88% stated that a psychiatric history is a risk factor for meditation-related challenges; 54% stated that a trauma history is important. Similarly, Lomas et al. (2015) found that pre-existing depression and anxiety could be exacerbated by Buddhist meditation. Several studies of MBPs suggest a relationship in the opposite direction; i.e., under some circumstances, participants with severe symptoms and traumatic backgrounds may be more likely to benefit. However, these studies have not included broad assessments of harm. We summarize the findings here.

#### 6.2.1 Participant vulnerabilities and response to standard MBPs

Several studies of MBCT for preventing depressive relapse have shown stronger benefits for participants with higher levels of various vulnerabilities, including earlier onset of depression, more previous episodes (Ma & Teasdale, 2004; Teasdale et al., 2000), unstable remission (Segal et al., 2010), and childhood trauma (Kuyken et al., 2015; Williams et al., 2014). In most of these studies, the participants with fewer vulnerabilities showed effects for MBCT that did not differ from TAU, placebo, or antidepressant medication (ADM). Several of these studies found that participants with fewer previous episodes had nonsignificantly higher relapse rates in MBCT than in TAU. Ma & Teasdale noted that the small sample made it unclear whether MBCT was causing harm for the fewer-episodes subgroup (increasing the likelihood of relapse) or was only unhelpful (providing no benefit). An individual patient data meta-analysis (Kuyken et al., 2016) combined these and other trials and found that in the larger sample (*N* > 1200) the only significant moderator of treatment effect was severity of depressive symptoms at baseline. Patients with more severe symptoms showed a larger treatment effect. No evidence of increased risk of relapse in MBCT was seen; however, asymmetry in the funnel plot suggested the possible existence of small unpublished studies showing this pattern.

Other MBPs have also shown better outcomes in participants with more severe symptoms. Roos, Bowen, and Witkiewitz (2017) found that MBRP was more effective than CBT or usual care for participants with high levels of substance use, anxiety, and depression, but similar to these comparison groups for participants with fewer symptoms. Arch and Ayers (2013) found that MBSR was more effective than CBT for people with comorbid anxiety and depression; CBT was more effective for those with anxiety only. These studies have not included detailed assessments of harm.

#### 6.2.2 MBPs adapted for specific vulnerabilities

MBPs have been adapted for psychosis, post-traumatic stress (PTS), and suicide risk. For people with psychosis, Chadwick, Taylor, and Abba (2005) reduced the number and duration of sessions, shortened the meditation practices, and provided steady guidance during practices to prevent absorption in psychotic symptoms during stretches of silence. Psychological functioning improved and no adverse effects were reported in post-treatment interviews. Since then, a meta-analytic review of MBPs for psychosis (Khoury et al., 2013) reported a small-to-moderate reduction in psychotic symptoms (g = 0.43) but did not discuss harm or adverse events.

MBPs for PTS have also been studied. As noted earlier, Banks et al. (2015) concluded that adverse effects in 12 studies of MBPs for PTS were minimal. A meta-analysis of 18 randomized trials (Hopwood & Schutte, 2017) found a mean effect size of g = 0.44 in favor of MBPs over control conditions. For studies of MBSR, rather than unspecified mindfulness programs, g = 0.49. There was no significant difference between MBSR adapted for trauma and standard MBSR. Most studies did not mention whether AEs had been examined; a few reported that there were none. One study reported that two participants (4.3%, one each in the MBP and the control condition) required hospitalization for worsening symptoms.

The adaptation of MBCT for people at high risk of suicide (Williams et al., 2014) found that MBCT was more effective than control conditions in preventing depressive relapse for people with a history of childhood trauma. Fifteen SAEs were reported: five in the MBCT arm and 10 in the control conditions (5% and 6.4% of participants, respectively). Most were overnight hospital admissions for physical health problems; 14 of the 15 events (93%) were judged to be unrelated to participation in the study. One episode of serious suicidal ideation was potentially attributable to the active control intervention (Williams et al., 2014).

Overall, the literature suggests that MBPs can be significantly therapeutic in groups with severe symptoms, comorbid conditions, and other vulnerabilities. Specific vulnerabilities are sometimes associated with better outcomes. Studies that monitored AEs/SAEs in these populations have reported that they occur in 0–10% of participants, are no more common in MBPs than control groups, and are not attributable to the MBP when they occur (Kuyken et al., 2016). However, many studies have not monitored harm in any way. When negative outcomes are reported, their relationship to participation in the MBP is not always provided. In addition, most studies report only group data, making it impossible to know whether promising averages mask deterioration in some participants.

### 6.3 Teacher/clinician factors

The literature summarized in Table 2 suggests that risk of harm may be related to characteristics of the provider, including empathy, understanding of the client's problems, communication about the nature of the program, skillful implementation of the program, managing difficulties that arise, and encouraging adherence to recommended practice. Here we summarize recent work on assessment of similar competencies in providers of MBPs.

#### 6.3.1 Assessment of teacher competencies

Several methods for assessing teachers' delivery of MBPs are available. All use ratings of recorded sessions or live observation and have shown adequate psychometric properties. The MBCT adherence scale (MBCT-AS; Segal, Teasdale, Williams, & Gemar, 2002) is a list of essential program elements; raters note the extent to which each element is present. The mindfulness-based relapse prevention adherence and competence scale (MBRP-AC; Chawla et al., 2010) is a similar measure assessing the presence of required elements and the therapist's skills in implementing them. The Mindfulness-Based Interventions – Teaching Assessment Criteria (MBI-TAC; Crane et al., 2013) evaluates six domains of competency: a) coverage, pacing, and organization of session content, b) relational skills, c) embodiment of mindfulness, d) guiding mindfulness practices, e) conveying course themes, and f) holding the group learning environment.

Relationships between teacher competency scores and participant outcomes have been examined in only a few studies. Using the MBRP-AC, Chawla et al. (2010) found that therapists' adherence to required elements of treatment was related to participants' development of mindfulness skills. In MBCT for adults with recurrent depression, Huijbers et al. (2017) found no significant relationships between teachers' MBI-TAC scores and any of the dependent variables. The null findings may be related to the small sample of teachers (*N* = 15), restriction of range in competence scores, or the high level of standardization of the intervention, which was provided in two clinical trials by the same research team.

While substantial progress has been made in the assessment of teacher competency, additional research is needed. To date, there is no evidence that lack of competency is related to harmful outcomes. Competency may have been assessed primarily in settings where teachers are well trained, leading to restriction of range. Prevention of harm may require skills not covered by existing measures, such as the pre-course interview. In ostensibly nonclinical settings such as workplaces, where MBPs may be offered by non-mental-health professionals, harm might arise when teachers lack skills for screening participants for psychological symptoms and managing mental health emergencies. Finally, because harm can arise through ethical violations, knowledge of professional ethics is necessary for managing issues related to informed consent, confidentiality, and other ethical concerns.

## 7 Recommendations for research

Perhaps the most basic unanswered questions are how often MBPs cause harm, in what forms, and to whom. Answering these questions requires detailed monitoring of potentially harmful effects. Surveys of participants about their experiences and measurement of a wide range of participant characteristics and outcomes might help to clarify whether the target problem got worse or new problems arose, and for whom. Attention to reasons for attrition may clarify whether dropping out is related to harm. Examining individual-level data could clarify whether group averages conceal the occurrence of harm in some participants. Vagueness in existing definitions of harm (how much deterioration is meaningful?) might be addressed with the reliable change index (Jacobson & Truax, 1991), which classifies participant outcomes into three categories: reliable improvement, no reliable change, or reliable deterioration. At the same time, it seems important to consider whether participants feel worse, even if their objectively measured symptoms have not reliably decreased (Duggan et al., 2014). Qualitative interviews with participants reporting harm or classified as having deteriorated may be helpful in generating hypotheses for quantitative studies.

More comprehensive monitoring may improve understanding of participant, teacher, and program factors potentially related to harm. For example, the study of moderators of outcome may clarify for whom programs should be modified or contra-indicated. If an MBP has both risks and benefits for many participants, research could focus on for whom the benefits outweigh the risks and how to mitigate risks. Harm that seems related to unskilful teaching may clarify essential teacher competencies. These factors are likely to be intertwined; that is, participant characteristics may require adaptations to programs and the development of specific teaching skills.

Consistent reporting of harm-related information in published papers is essential to developing this body of knowledge. A revised CONSORT checklist (Ioannidis et al., 2004) includes 10 recommendations for reporting on harm-related issues in randomized trials. These include listing and defining all adverse events that were studied, noting whether events that occurred were anticipated or unexpected, clarifying how harms-related information was collected and analyzed, presenting risk of each adverse event for each arm of the trial, and providing a balanced discussion of benefits and harms. Despite the longstanding availability of this checklist, such reporting is rare, for reasons that are unclear. Peterson et al. (2013) suggested that the primary reason is that a temporary increase in symptoms or discomfort is understood to be part of the normal therapeutic process. However, they also argued that greater reporting of this phenomenon would increase understanding of the overall risk and safety of therapies, especially those known to involve significant discomfort such as exposure-based treatments. More consistent reporting might also help to clarify the boundary between expected discomfort and potential harm.

Another reason for lack of reporting of harms may be overreliance on definitions of AEs and SAEs used in medical research (Duggan et al., 2014). Some of these events (suicide attempts, hospitalizations) may be uncommon in trials of psychological interventions or MBPs. Monitoring events more relevant to the program being studied would be informative. Duggan et al. (2014) also note that many adverse events are not spontaneously reported and will not come to light without systematic assessment methods such as structured interviews.

Several additional questions seem important for future research. First, what is the dose/response relationship for mindfulness practice in MBPs? Can high doses be harmful? To date, the literature has examined only whether extent of self-reported home practice predicts positive outcomes; a meta-analytic review (Parsons, Crane, Parsons, Fjorback, & Kuyken, 2017) found a small but significant association. This review did not report whether any of the correlations between home practice and outcome were negative and did not address the issue of harm.

Second, a related issue is whether the type, timing and quality of home practice influences outcomes and whether specific ways of practicing might cause harm. Lomas et al. (2015) found that practitioners of Buddhist meditation who used advanced practices before they were ready for them experienced difficult effects. Lindahl et al. (2017) referred to “incorrect ways of practicing meditation” (p. 23) that might cause harm; these are not clearly explained but include excessive striving, attachments to specific states, and misunderstanding or not following directions. For MBPs, Del Re, Fluckiger, Goldberg, and Hoyt (2013) developed a self-report measure of practice quality and found that symptom reduction was related to the extent to which participants returned their attention to present-moment experiences with curiosity, willingness, and self-compassion. However, they did not address harmful effects.

Third, if harm occurs, how can it be remediated? In medicine and exercise science, more is known about treatment of overdoses, adverse reactions, and injuries; an analogous body of knowledge is needed for the mindfulness field. Lindahl et al. (2017) include discussion of potential remedies for challenges arising in Buddhist meditation; however, little is known about how well these apply to difficulties arising in evidence-based MBPs.

## 8 Recommendations for protecting participants in MBPs from harm

Several steps can be taken before an MBP begins. Teachers must understand the theoretical and empirical foundations for using the MBP with their population. Without this understanding, they will be unable to explain to potential participants how the MBP may be relevant to them, what difficulties might arise, how these can be managed, and whether the potential benefits are likely to outweigh the difficulties (Kuyken, Crane, & Williams, 2012). This information should be part of a pre-program orientation and informed consent process in which the theoretical rationale, evidence base, and potential benefits and risks are discussed.

Careful assessment of potential participants and well considered exclusion criteria are important. Available lists of recommended exclusion criteria for MBPs (Kuyken et al., 2012; Santorelli et al., 2017) generally include substance dependence, suicidality, psychosis, PTSD, severe depression, severe social anxiety, and recent bereavement, divorce, or other personal crisis. Such conditions are likely to interfere with ability to participate in the group or the practices, or to receive any benefit from doing so. However, because this is not true in every case, these criteria are “subject to clinical judgement and experience of teacher, and support available to and motivation of participant” (Kuyken et al., p. 23). When working with the general population, assessing psychiatric and trauma history as well as current functioning and professional and personal support may facilitate sound decisions about readiness to participate and the need for concurrent psychological or psychiatric treatment (Dobkin, Irving, & Amar, 2012). As noted earlier, MBPs adapted for conditions that typically appear on lists of exclusion criteria (PTSD, suicidality, psychosis) have shown promising results, suggesting that exclusion criteria must be viewed flexibly.

Once the program begins, it is important to teach both the *what* and the *how* elements of mindfulness and to be sure that the psychoeducational and structural supports described earlier are in place. Rationales for the practices should be made clear and participants should feel invited, rather than pressured, to engage in them. Distress and discomfort are likely to arise as participants learn new skills and practice applying them to the difficulties for which they sought help. Prevention of harm requires understanding common types of uncomfortable experiences, their usual range of intensity, and how to help participants respond to them in ways that facilitate learning the desired skills. Systematic monitoring and recording of deterioration and adverse events will increase this knowledge in providers of MBPs.

Systematic monitoring may also help teachers to recognize when unusual or unexpected distressing experiences have arisen and when they are disproportionate to the expected benefits, likely to interfere with attaining benefits, or require clinical intervention. Protocols for responding to objective indicators of imminent harm have been used in randomized trials for monitoring foreseeable risks; for example, referral to a physician when a participant endorses suicidality on a questionnaire. While suicide risk is foreseeable in a depressed sample, other potentially harmful outcomes may be harder to predict. The Office for Human Research Protections (US Department of Health and Human Services, 2007) notes that an event is unexpected if its nature, severity, or frequency is inconsistent with the known or foreseeable risk associated with the procedures involved, or if it is inconsistent with the expected natural progression of an underlying condition. When such events occur, prevention of harm may require adjustments in the participant's practice, discontinuing the program, or referral to other services. Mindfulness teachers need training in the mental health conditions they are likely to encounter and how to recognize and work with the meditation-related challenges that participants may experience. The requirement that teachers of MBPs have their own mindfulness practice may provide further experiential understanding of challenging mind states that will help them work with participants' meditation-related challenges.

## 9 Summary and conclusions

In well-established approaches to health and wellbeing, including psychotherapy, pharmacotherapy, and physical exercise, some participants suffer serious harm or get meaningfully worse. The same appears to be true for meditation in contemplative traditions. Evidence-based MBPs have important commonalities with these approaches. They work with cognitions, emotions, and sensations, some of which are distressing and difficult; they raise issues about adherence, dosage, and dose/response relationships; they include a variety of exercises, which can be uncomfortable; and they place the formal practice of meditation at their core. Because of these commonalities, it is essential to consider the possibility that some participants in evidence-based MBPs may get worse.

The existing literature on harm in evidence-based MBPs is sparse. A few studies have shown worsening symptoms in MBPs; however, meta-analyses consistently report significant benefits for many outcome variables in a wide range of samples. Research also suggests that participants with severe symptoms, comorbid conditions, and other vulnerabilities (psychosis, trauma history, suicide risk) can benefit from MBPs in standard or adapted forms, and that some may show more benefit from MBPs than participants without such vulnerabilities. The few reviews including data on AEs and SAEs in evidence-based MBPs report that they have occurred in zero to 10.6% of participants (Table 4), are no more common in MBPs than comparison conditions (Kuyken et al., 2016; Wong et al., 2018), and are not attributable to the MBP (Kuyken et al.) or not clinically significant (Banks et al., 2015).

However, these findings must be viewed cautiously because most studies report only group averages that might mask meaningful deterioration in some participants. Only a small minority of studies have monitored AEs and SAEs. Those that have may have defined them narrowly or failed to ask about them in ways that elicit detailed answers.

The prevalence of harm from meditation in contemplative traditions is unclear. Nevertheless, harm clearly occurs, and the possibility that similar harm might arise in evidence-based MBPs, despite adaptations for contemporary mainstream contexts and the presence of structural and psychoeducational supports described earlier, needs further study. If such harm is occurring, it might not be detectable without systematic monitoring of individual-level data. The ethical obligation to do no harm requires us to enhance our monitoring methods in order to better understand the risks for participants in MBPs, including what forms of harm might occur, how often they occur, who is most susceptible, and how harmful effects can be prevented or remediated.

## Role of funding sources

This work was partially supported by the Wellcome Trust, Grant Reference: 104908/Z/14/Z. The Wellcome Trust had no role in planning or writing the manuscript or the decision to submit the paper for publication.

## Contributors

All authors contributed to discussions about the goals and structure of the paper. Ruth Baer wrote most of the first draft. Catherine Crane, Edward Miller, and Willem Kuyken wrote sections of the paper. All authors contributed to editing and revising. All authors have approved the final manuscript.

## Conflict of interest

Ruth Baer is an Associate of the Oxford Mindfulness Centre and receives occasional payments for training workshops and presentations related to mindfulness. She also receives royalties for several books related to mindfulness. Catherine Crane is affiliated with the Oxford Mindfulness Centre and funded by the Wellcome Trust on a strategic award exploring the role of mindfulness training in adolescence. However she does not receive additional remuneration for training workshops or presentations related to mindfulness. Willem Kuyken is the director of the Oxford Mindfulness Centre. He receives payments for training workshops and presentations related to mindfulness and donates all such payments to the Oxford Mindfulness Foundation, a charitable trust that supports the work of the Oxford Mindfulness Centre. Willem Kuyken was until 2015 an unpaid Director of the Mindfulness Network Community Interest Company and gave evidence to the UK Mindfulness All Party Parliamentary Group.
